# Microbiota of newborn calves and their mothers reveals possible transfer routes for newborn calves’ gastrointestinal microbiota

**DOI:** 10.1371/journal.pone.0220554

**Published:** 2019-08-01

**Authors:** Daniela Klein-Jöbstl, Narciso M. Quijada, Monika Dzieciol, Benjamin Feldbacher, Martin Wagner, Marc Drillich, Stephan Schmitz-Esser, Evelyne Mann

**Affiliations:** 1 Department for Farm Animals and Veterinary Public Health, Clinical Unit for Herd Health Management, University Clinic for Ruminants, University of Veterinary Medicine Vienna, Vienna, Austria; 2 Laboratory of Molecular Biology and Microbiology, Instituto Tecnológico Agrario de Castilla y León, Valladolid, Spain; 3 Department for Farm Animals and Veterinary Public Health, Institute of Milk Hygiene, Milk Technology and Food Science, University of Veterinary Medicine Vienna, Vienna, Austria; 4 Department of Animal Science, Iowa State University, Ames, Iowa, United States of America; Wageningen University, NETHERLANDS

## Abstract

The intestinal microbiota of newborns plays an important role in the development of immunity and metabolism. In livestock animals, knowledge of the intestinal microbiota is essential not only to prevent diseases but also to optimize weight gain and performance. The aim of our study was to examine faecal samples repeatedly within the first two days of life using 16S rRNA gene High Throughput Sequencing. Additionally, samples from the mouths of the calves and the vaginas, colostrum, and faeces of the dams were included to evaluate possible sources of the calf faecal microbiota. The calf faecal microbiota was highly variable during the first 48 hours *post natum* (*p*.*n*.). Significant changes were found in species diversity and richness, in copy numbers evaluated by qPCR and in predominant bacteria over time. The most pronounced changes occurred between 6 and 24 hours *p*.*n*. All calf faecal samples were dominated by Operational Taxonomic Units (OTUs) belonging to the family *Enterobacteriaceae*. Cow faecal samples showed significantly higher species richness, diversity, number of observed OTUs, and copy numbers compared to all other samples. OTUs belonging to the family *Ruminococcaceae* were most abundant in cow faecal and vaginal samples. Colostrum was dominated by *Enhydrobacter* affiliated OTUs. To identify possible inoculation routes for the calf microbiota, we analysed OTU sharing between samples. The calf microbiota during the first two days of life was clearly distinct from the dam’s faecal microbiota. Furthermore, colostrum microbiota clearly differed from calf and cow faecal microbiota and thus most likely does not play an important role as inoculation source for calf microbiota during the first two days of life. In contrast, the cow vaginal and the calf faecal microbiota were more similar, suggesting that some of the calf faecal microbiota may derive from inoculation from the birth canal during birth.

## Introduction

Birth exposes the newborn to the vaginal flora of the mother and the environment, setting in motion the bacterial colonisation of the gastrointestinal tract [1]. Up to date, there are few studies available that examined the intestinal and faecal microbiota in newborn calves [2, 3]. Some recent studies have analysed the development of microbial communities in calves from birth to one or up to three weeks of life, weaning and/or to adulthood [2–9]. However, most of these studies have sampled during later stages of calf development and not during the first days or even hours of life [10]. In all studies, microbiota composition varied between sampling time points and sampling sites with significant changes in α-diversity in gut-related samples over the first weeks of life.

The intestinal microbiota has an important effect on the maturation of the adaptive and innate immune system during early development [11]. Furthermore, the gastrointestinal microbiota has important metabolic and nutritional effects [12]. Consequently, the intestinal microbiota influences the animals’ health and performance and has a great impact on the overall welfare [1, 13]. Hence, knowledge of early bacterial colonisation and their possible sources may help to improve calf management during the first days of life.

The aim of our study was to examine the colonisation of the intestinal tract of newborn calves within the first two days of life by analysing faecal samples using High Throughput Sequencing of 16S rRNA gene amplicons. Furthermore, calf mouth samples as well as maternal vaginal, colostrum, and faecal samples were included to evaluate possible sources of the calves`faecal microbiota and to define a core microbiota in these samples.

The hypothesis of the study was that in newborn calves the intestinal tract is colonised by bacteria quickly after birth. According to the literature, we hypothesized that the initial microbiota of the newborn calf is influenced by the microbiota of the birth canal, the dam’s faecal microbiota, and the colostrum fed.

## Materials and methods

The study was approved by the institutional ethics and animal welfare committee of the University of Veterinary Medicine, Vienna (ETK-03/05/2015), as well as by the Slovakian Regional Veterinary Food Administration.

### Study design and sampling procedure

**Fig 1:**
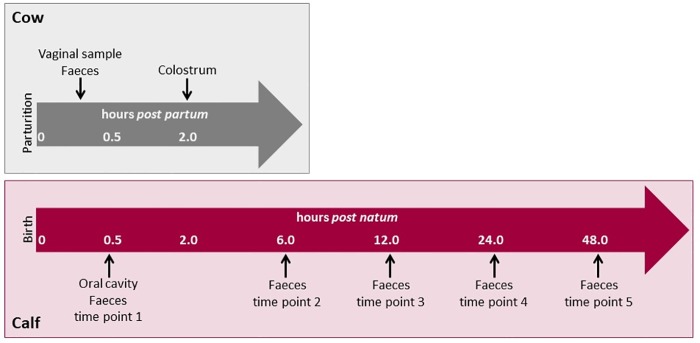
Overview over samples and sampling time points in regard to parturition and birth in cow-calf pairs.

Overall, 15 Holstein-Friesian calves and their mothers were included in the study. To reduce environmental, management, and seasonal bias, the study was performed on one commercial dairy farm within two weeks. All animals examined within the study were part of the commercial dairy herd, born and reared on farm. All cows calved in a group calving pen under permanent supervision of a farm worker. The calf was removed from the dam and brought to a clean individual pen immediately after birth. Afterwards the cow was fixed for routine treatment and colostrum harvesting. Colostrum was fed to the calves approximately two hours *p*.*n*. Only healthy calves delivered vaginally without assistance were enrolled in the study. All animals were sampled by the same experienced veterinarian (DKJ). Samples were taken from the cows`vagina immediately after parturition, when the cow was fixed. The samples were taken by use of a sterile cytobrush (Gynobrush, Heinz Herenz, Hamburg, Germany; 20 mm in length and 7 mm in diameter) as described by Prunner et al. [14]. In short, the cytobrush was screwed on a metal rod of 65 cm length. The brush was protected by a disposable plastic catheter and plastic sleeve. Before sampling the vulva was cleaned with dry paper. The cytobrush was inserted, protected by a hand using a sterile glove. Inside the vagina, the plastic sleeve was retracted and the brush moved forward carefully and rolled along the cervical wall. The brush was drawn back into the catheter before the instrument was removed from the reproductive tract. Two samples were taken from each cow. The cytobrush was then stored in a sterile tube with 1.5 ml RNAlater solution (ThermoFisher Scientific). Smears from the calf mouth were also taken by cytobrush after the calf was moved to the individual pen (within 0.5 hours *p*.*n*.), before first colostrum feeding. The cytobrush was inserted into the mouth, rolled over the buccal cavity and afterwards placed in a sterile tube with RNAlater solution. Immediately before first colostrum feeding, 50 ml of colostrum was taken of the bucket fed to the individual calf and stored in a sterile tube. All faecal samples were taken from the rectum by use of a sterile glove and lubricant. A minimum of 1g faeces per sampling time was taken. The dams were sampled once (immediately after parturition and sampling of the vagina), whereas five faecal samples were taken from calves within 0.5 hours (before first colostrum feeding), and at 6, 12, 24 and 48 hours *p*.*n*. (Fig 1). All samples were immediately frozen at -20°C on farm and shipped frozen to the laboratory of the University of Veterinary Medicine Vienna for further processing.

### Sample preparation and DNA extraction

The sample collection included colostrum (n = 15), calf faeces at five time points (n = 74), cow faeces (n = 15), calf mouth (n = 10) and cow vagina (n = 15). In total, these 129 samples and one negative process control were applied to sample preparation and DNA isolation. Sample preparation was applied prior DNA isolation to optimise microbial loads and 16S rRNA gene PCR amplifications. The optimized sample preparation of each sample group was as follows:

Colostrum samples (10 ml each) were centrifuged at 8,000×g for 5 min at 4°C and the supernatant, including the fat cream layer, was discarded. The obtained pellet was washed thrice in 1×PBS (phosphate buffered saline; pH 7.3) and centrifuged again at each step at 8,000×g for 5 min at 4°C. The obtained pellet was applied to the DNA isolation.

For the smear samples from the calf mouths and cow vaginas, the cells retrieved on the cytobrush, which had been stored in 1.5ml RNAlater solution, were submerged in up to 5 ml of sterile Ringer solution. Samples were vigorously horizontally agitated (5 min) to dislodge cells from the cytobrush by using the MOBIO Vortex adapter tube holder for the vortex. The cells were pelleted by centrifugation at 12,000×g for 10 min at 4°C. After that, the cytobrush was carefully removed, and the centrifugation step was repeated. The pellet was applied to the DNA extraction.

For cow faeces, a sample preparation protocol was not necessary, and extraction of genomic DNA was performed by using 220mg of thawed, homogenized wet faeces per cow. DNA was extracted from all samples using QIAamp DNA Stool Mini Kit (QIAGEN, Hilden, Germany) following the recommendations of the manufacturer. For calf faeces samples, the DNA yield was increased by pooling multiple extraction eluates. For time point 0.5, 6 and 12h *p*.*n*., DNA was extracted in triplicate from 220mg of thawed, homogenized, wet faeces per calf. For time points 24 and 48 hours *p*.*n*., DNA was extracted in duplicate. For each calf sample, a total of 600 μl or 400 μl DNA suspension (200 μl from each 220 mg aliquot) was concentrated by ethanol precipitation. The DNA pellet was re-suspended in 100 μl ddH_2_O.

### 16S rRNA gene amplicon sequencing

Extracted DNA samples (n = 129) and a negative process control were used for 16S rRNA gene PCRs (V345) using the primers 341F (5’-CCTACGGGRSGCAGCAG-3’) and 909R (5’-TTTCAGYCTTGCGRCCGTAC-3’) including the universal 5’ tails as specified in the Nextera library protocol from Illumina. The amplicons (approx. 568bp) were sent to Microsynth, Balgach, Switzerland. Libraries were constructed by ligating sequencing adapters and indices onto purified PCR products using the Nextera XT Sample Preparation Kit (Illumina) according to the recommendations of the manufacturer. Equimolar amounts of each of the libraries were pooled and submitted for sequencing on an Illumina MiSeq platform using a 300 bp read length paired-end protocol.

### Sequence analysis

Quality of 16S rRNA gene amplicons sequences was evaluated by using FASTQC (http://www.bioinformatics.babraham.ac.uk/projects/fastqc/). Based on a Phred score below 20 within a 20-bp-long window, reads were trimmed, and reads shorter than 150 bp were discarded by using Prinseq [15]. Paired-end reads were then merged by using FLASH [16]. Chimeric sequences were excluded by using the “gold” database (https://drive5.com/uchime/uchime_download.html), and singletons were excluded. In order to avoid biases due to different sequencing depths, all samples were rarefied to 23,030 reads per sample. Sequences were then analyzed by using QIIME v1.9.1 [17]. Operational taxonomic units (OTUs) defined by a 97% similarity threshold were picked using the UCLUST algorithm [18]. The most abundant sequence in each OTU was chosen as its representative sequence. The RDP naïve classifier [19] was used to assign the taxonomy of the representative sequence of each OTU against the GreenGenes 16S rRNA database [20] by using the assign_taxonomy.py script. The number of reads assigned to each OTU was calculated by using the make_otu_table.py script. Alpha (Good’s coverage, Chao1, Shannon and Simpson indices) diversity measures and Weighted UniFrac [21] distance matrices were obtained through QIIME. The OTU table filtered at 0.01% abundance was used to generate a bipartite graph by using QIIME (“*make_otu_network*.*py*”). Cytoscape 3.3.0 was used to visualize OTU networks [22]. Nodes represent either samples or bacterial OTUs. Connections were drawn between OTUs and the samples they belong, with edge weights defined as the number of sequences from each OTU that occurred in each sample.

### Quantitative PCR

For quantification of bacterial 16S rRNA gene copy numbers, standard curves were constructed by using the primer set 341F (5′-CCT ACG GGA GGC AGC AG-3′) and 534R (5′- ATT ACC GCG GCT GCT GG -3′) to amplify serial dilutions of purified PCR products from all sample types as recently described by Metzler-Zebeli et al. [23]. Copy numbers of standard curves were calculated using the following equation: DNA (molecules/μL) = [6.02 × 1023 (molecules/mol) × DNA amount (g/μL)]/[DNA length (bp)× 660 (g/mol/bp) x109 ng/g]. For the standard template 4.83 × 10^9^ copies per ng were calculated.

Real-time quantitative PCR reactions were run in duplicate (final volume of 25 μL) using MicroAmp 0.2 mL optical tubes sealed with MicroAmp optical 8-cap strips (Applied Biosystems, Foster City, CA, USA). A single amplification reaction consisted of 12.2 μL diethylpyrocarbonate (DEPC)-treated water, 2.5 μL 10×buffer, 1.5 μL 3 mM MgCl_2_ (stock concentration 50mM), 1 μL of each primer (stock concentration 10 μM), 0.5 μL undiluted EvaGreen fluorescent DNA stain (JenaBioscience, Jena, Germany), 1 μL of dNTP Mix (stock concentration 20 mM, 5 mM of each dATP, dCTP, dGTP and dTTP; Thermofisher, Vienna, Austria), 0.3 μL of Platinum Taq DNA polymerase (5 U/μl; Thermo Fisher Scientific, Vienna, Austria), and 5 μL template (genomic DNA). The quantification of DNA was performed in a Mx3000P qPCR instrument (Stratagene, La Jolla, CA, USA) (software v.4.10) after initial denaturation at 94°C for 2 min, followed by 45 cycles of 94°C for 30 s, 60°C for one min. To determine the specificity of the amplifications, dissociation curves after each reaction were recorded and carried out at 95°C for one min, followed by complete annealing at 60°C for 30 s and a gradual increasing temperature up to 95°C.

The final copy numbers of total bacteria were calculated using the quantitative mean of the copy number per ml or g, including calculation of the DNA volume subjected to qPCR, the volume of extracted DNA, and the weight or volume of the sample subjected to DNA extraction. Additionally, an average of four 16S rRNA gene copies per genome was taken into account when extrapolating the final copy numbers [24].

### Statistical analyses

Differences in relative abundances on phylum and OTU level as well as in species richness and diversity indices and results of qPCR between calf faecal samples of the different sampling time points and other samples (calf mouth, cow vagina, colostrum, and faeces) were examined by paired Wilcoxon test with Benjamini-Hochberg correction. Statistical analysis were performed using SPSS Statistics for Windows (version 24.0; IBM Deutschland GmbH, Ehningen, Germany). The level of significance was set at *P*<0.05.

## Results

Sequencing of the negative process control resulted in 209 reads. After quality filtering, paired-end joining and chimera removal of the raw sequencing data, 5,080,635 reads remained (41,081 ± 14,517 reads per sample) ranging from 23,030 to 114,596 reads per sample. Eleven samples (five calf faeces, four cow vagina, and two calf mouth samples) did not pass the initial quality control, and thus they were removed from the sequence analysis. Sequences were classified into 11,905 OTUs using a 97% sequence similarity threshold. Of these, 113 OTUs showed an abundance higher than 0.1%, and 9 showed an abundance higher than 1.0%. The most abundant OTU (OTU-1 unclassified *Enterobacteriaceae*) accounted for 23.5% of the reads and was present in all of the 118 samples studied. Twenty-one OTUs, representing 0.44% of all reads, were assigned to *Archaea*.

### Calf faecal microbiota during the first 48 hours of life

**Fig 2:**
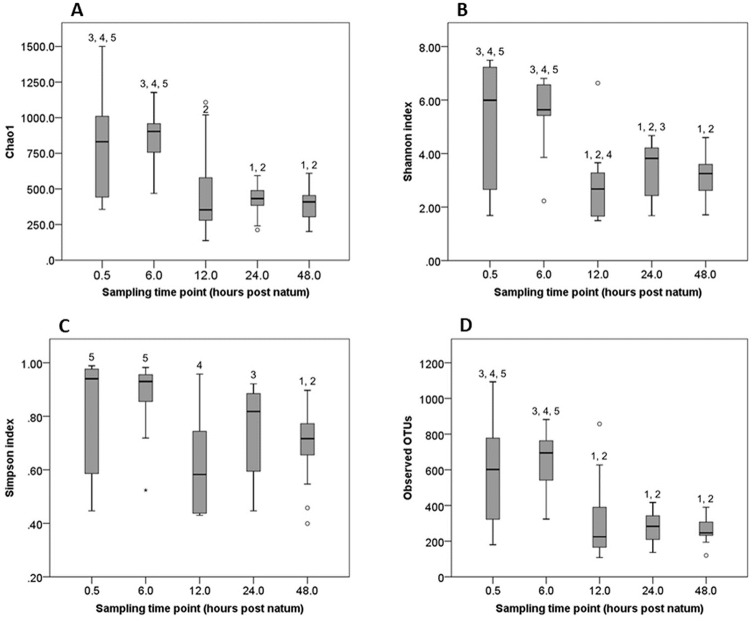
(A) Species richness (Chao1), (B) Shannon and (C) Simpson diversity index and (D) number of observed OTUs in calf faeces during the five sampling time points. Data are visualized as box-and-whisker plots showing the median and the interquartile (midspread) range (boxes containing 50% of all values), the whiskers (representing the 25 and 75 percentiles) and the extreme data points. Numbers above boxes indicate significant differences to the time points.

Median good`s coverage for the five sampling time points varied between 99.2 and 99.6%. Species richness (Chao1), diversity (Shannon and Simpson diversity index), and the number of observed OTUs significantly differed between calf faecal samples of different time points. Generally, a significant decrease between early time points 1 and 2 (0.5 and 6 hours *p*.*n*.) and later time points 3 to 5 (12 to 48 hours *p*.*n*.) was found (Fig 2).

The OTUs affiliated to 30 bacterial phyla with *Proteobacteria*, *Firmicutes*, *Actinobacteria*, and *Bacteroidetes* being the most abundant. *Proteobacteria* were most abundant in calf faecal samples during all sampling time points with a median relative abundance ranging from 71.9 to 91.2% (S1 Fig). *Actinobacteria* and *Bacteroidetes* decreased from time points 1 and 2 (0.5 and 6 hours *p*.*n*.) to time point 3 (12 hours *p*.*n*.) and remained low through the other time points (24 and 48 hours *p*.*n*.). *Proteobacteria* increased from time point 1 to 3, and *Firmicutes* increased from time point 3 to 5.

Calf faecal samples were clearly dominated by *Gammaproteobacteria* OTUs with *E*. *coli / Shigella* and *Acinetobacter* OTUs being most abundant. At all five sampling time points, OTU-1 (unclassified *Enterobacteriaceae*) was most abundant, with a median relative abundance varying between 26.0% during time point 2 and 59.0% during time point 3. The overall median relative abundance was 40.0%. The 50 most abundant OTUs in calf faecal samples accounted for 62.7 and 58.2% of all reads during time points 1 and 2 and for more than 90% of all reads during time points 3 to 5.

**Fig 3:**
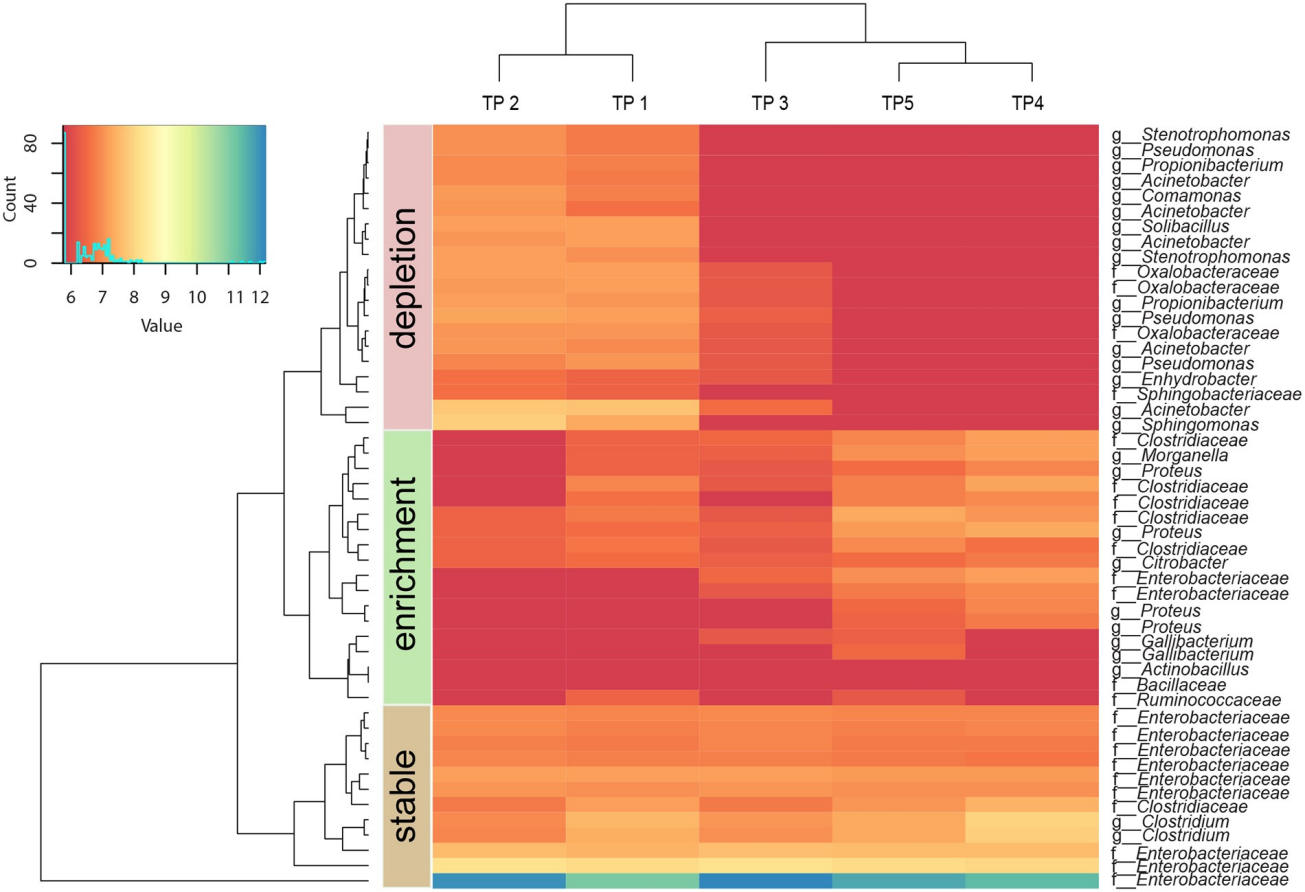
The 50 most abundant OTUs in calf faecal samples and time-related abundance shifts. The dendrogram was built based on Euclidean measures and was used to define the abundance clusters ‘stable’ (stable abundance), ‘enrichment’ (abundance increase over time) and ‘depletion’ (abundance decrease over time). TP = time point, TP 1 = 0.5 hours *post natum* (*p*.*n*.), TP 2 = 6 hours *p*.*n*., TP 3 = 12 hours *p*.*n*., TP 4 = 24 hours *p*.*n*., TP 5 = 48 hours *p*.*n*.

The majority (≥ 70%) of the top 50 OTUs over all calf faecal samples differed significantly between time points 1 and 2 and between later time points 4 and 5. Several OTUs (e.g. OTU-2 *Enhydrobacter*, OTU-4 *Acinetobacter indicus*, OTU-11 *Sphingomonas*, OTU-18 *Acinetobacter*, OTU-19 *Solibacillus*, OTU-28 *Pseudomonas*, OTU-30 *Acinetobacter lwoffii*, OTU-31 unclassified *Ruminococcaceae*, OTU-32 unclassified *Clostridiales*, OTU-33 *Propionibacterium acnes*, OTU-34 unclassified *Ruminococcaceae*, and OTU-47 *Stenotrophomonas*) were only found during the first two to three sampling time points, and others were found only during later time points 4 and 5 (e.g. OTU-14 *Clostridium butyricum*, OTU-16 unclassified *Enterobacteriaceae*). In contrast, there were few significant differences when comparing time point 1 with time point 2, and comparing time point 4 with time point 5. This indicated a change in the most abundant OTUs between 6 and 24 hours *p*.*n*. (Fig 3).

Copy numbers, obtained by qPCR using a general 16S rRNA gene primer set, revealed a significant 115-fold increase in copy numbers during late time points (24h and 48h *p*.*n*., median: 5.84×10^6^copy numbers) compared with earlier time points (median: 5.09×10^4^copy numbers) (Table 1).

**Table 1:** Results of qPCR for calf faecal samples (along the time line).

	Calf faeces sampling time point (TP*)				
	TP 1	TP 2	TP 3	TP 4	TP 5
Median	8.74×10^3^	1.08×10^5^	3.60×10^4^	5.77×10^6^	5.91×10^6^
Percentile 25%	4.26×10^3^	2.53×10^4^	5.64×10^3^	2.37×10^6^	2.83×10^6^
Percentile 75%	1.37×10^5^	2.16×10^5^	7.59×10^5^	1.40×10^7^	2.78×10^7^
Minimum	2.24×10^2^	1.08×10^3^	5.22×10^2^	1.44×10^4^	2.62×10^5^
Maximum	1.61×10^6^	5.25×10^5^	2.00×10^7^	2.16×10^7^	5.60×10^7^
Significantly different to TP*	4, 5	4, 5	4, 5	1, 2, 3	1, 2, 3

Descriptive statistics and results of paired Wilcoxon test with Benjamini-Hochberg correction (*P*<0.05) are given.

*TP 1 = 0.5 hours post natum (*p*.*n*.), TP 2 = 6 hours *p*.*n*., TP 3 = 12 hours *p*.*n*., TP 4 = 24 hours *p*.*n*., TP 5 = 48 hours *p*.*n*.

### Microbiota of the cow (vagina, faeces, and colostrum)

Significantly higher species richness, diversity, and number of observed OTUs were detected in cow faecal samples when compared to all other samples.

The composition of microbial communities on phylum level revealed distinct differences between the different sampling sites (S1 Fig). *Firmicutes* were most abundant in cow vaginal and faecal samples (median relative abundance 63.4 and 93.7%, respectively) whereas colostrum was clearly dominated by *Proteobacteria* with a median relative abundance of 84.9%.

In cow faeces a high inter-individual similarity was seen. Overall, 382 OTUs were shared by all 15 cow faecal samples. These OTUs accounted for 77.4% of all reads in these samples. In contrast, vaginal and colostrum samples shared 17 and 32 OTUs (accounting for 9.8 and 64.1% of all reads), respectively. OTUs belonging to the family *Ruminococcaceae* were most abundant in cow vaginal and especially faecal samples with a median relative abundance of 59.1 and 84.8%. Colostrum was dominated by *Enhydrobacter* affiliated OTUs (overall median relative abundance 80.5%; S1 Table).

Copy numbers obtained by qPCR revealed significant higher counts in cow faeces compared with all other sampling sites (>1.39×10^8^ copy numbers). Colostrum, cow vagina, and calf mouth copy numbers ranged from 3.24×10^3^ to 1.17×10^6^. Exact values are listed in Table 2.

**Table 2:** Copy number results of qPCR for cow faeces, cow colostrum, cow vagina, and calf mouth samples.

	Cow faeces	Colostrum	Cow vagina	Calf mouth
Median	5.00×10^8^	4.55×10^5^	2.95×10^4^	1.27×10^4^
Percentile 25%	2.72×10^8^	1.34×10^5^	2.19×10^4^	7.06×10^3^
Percentile 75%	6.34×10^9^	1.17×10^6^	2.88×10^5^	2.38×10^4^
Minimum	1.39×10^8^	6.43×10^4^	8.14×10^3^	3.24×10^3^
Maximum	8.32×10^9^	2.30×10^6^	9.41×10^5^	2.88×10^4^

Results of descriptive statistics are given. Paired Wilcoxon test with Benjamini-Hochberg correction revealed a significant difference (*P*<0.05) between all four sampling sites.

### OTUs specific for cow or calf samples

Among the 50 most abundant OTUs over all samples, we identified some OTUs which were only found in cow samples. These included OTU-10 (*Gallibacterium*) and OTU-31 (unclassified *Ruminococcaceae*), which were found only in cow fecal samples, as well as colostrum-associated OTUs such as OTU-12 (*Meiothermus*), OTU-37 (*Enhydrobacter*), OTU-44 (*Chryseobacterium*), or OTU-50 (*Sphingobacterium faecium*). None of these OTUs were detected in cow vaginal samples. In contrast, OTU-5 (*Clostridium*), OTU-8 (*Clostridium perfringens*), OTU-9 (*Proteus*), OTU-14 (*Clostridium butyricum*), OTU-17 (unclassified *Clostridiaceae*), OTU-15 (*Morganella*), and OTU-16 (unclassified *Enterobacteriaceae*) were exclusively detected in calf samples.

**Fig 4:**
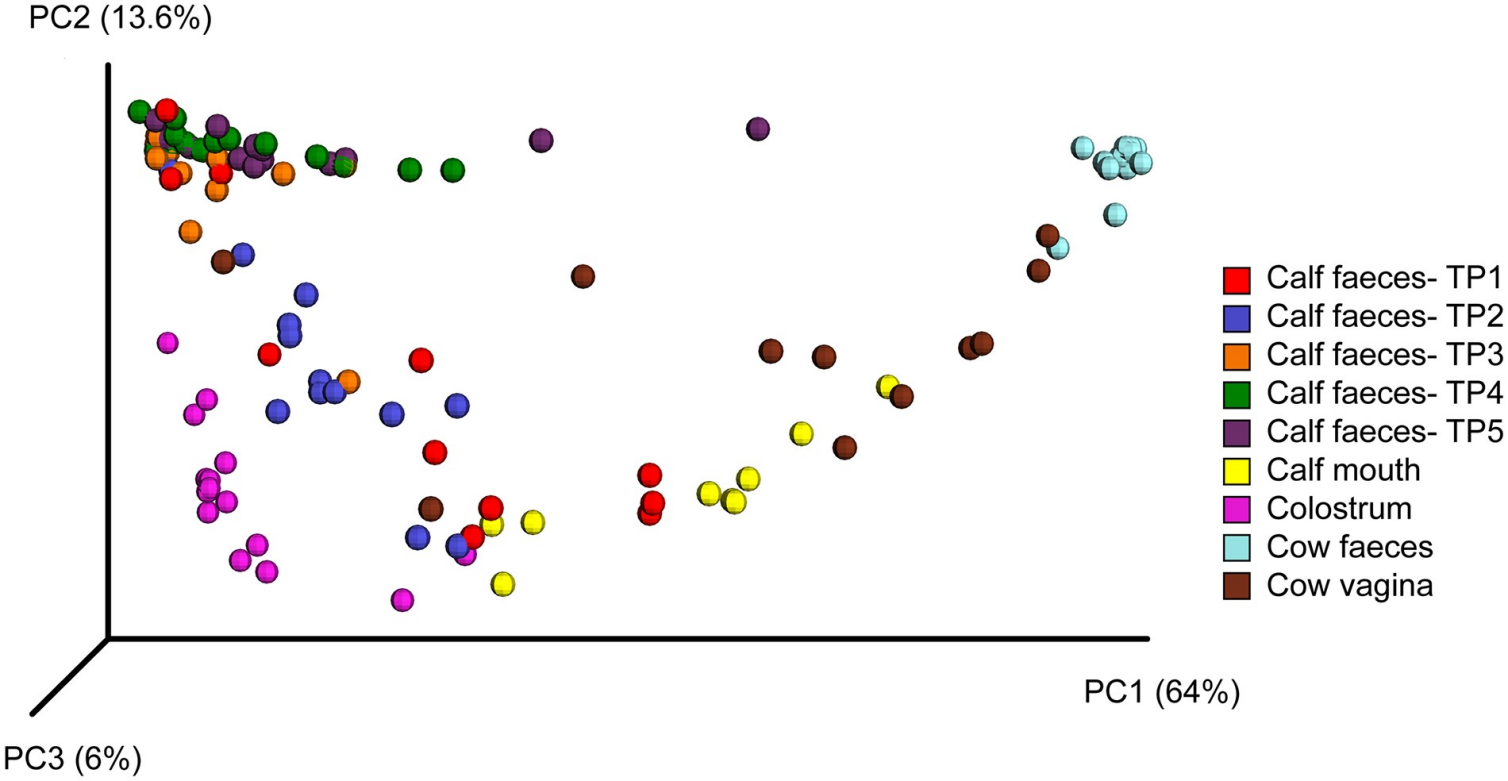
Principal Coordinate Analysis (PCoA) of 16S rRNA gene OTUs based on Weighted UniFrac distances. Dots are colored regarding the sample type. TP = time point, TP 1 = 0.5 hours *post natum* (*p*.*n*.), TP 2 = 6 hours *p*.*n*., TP 3 = 12 hours *p*.*n*., TP 4 = 24 hours *p*.*n*., TP 5 = 48 hours *p*.*n*.

Weighted UniFrac distances were calculated and represented as a principal coordinates analysis (PCoA, Fig 4). In contrast to the high inter-individual similarity between cow faecal samples, all other samples showed considerable differences. Cow faeces that showed a high similarity within the group clustered together. Calf faecal samples shifted throughout the time. Time points 1 and 2 did not cluster together, and samples show similarity to calf mouth and colostrum. Later time points clustered, indicating a stabilisation of faecal microbiota at later time points.

### Possible sources of the newborn calf faecal microbiota during the first 48 hours of life

To evaluate possible sources of the newborn calf faecal microbiota, vaginal samples, cow faecal samples, colostrum, and the calf mouth were examined as possible sources.

Thirteen of the 50 most abundant OTUs were shared between cow vaginal and calf faecal samples: OTU-4 (*Acinetobacter*), OTU-6 (unclassified *Enterobacteriaceae*), OTU-7 (unclassified *Ruminococcaceae*), OTU-19 (*Solibacillus*), OTU-20 and OTU-21 (unclassified *Ruminococcaceae*), OTU-22 (unclassified *Enterobacteriaceae*), OTU-28 (*Pseudomonas*), OTU-30 (*Acinetobacter lwoffii*), OTU-32 (unclassified *Clostridiales*), OTU-33 (*Propionibacterium acnes*), OTU-46 (unclassified *Enterobacteriaceae*), and OTU-48 (*Comamonas*). These OTUs were mainly shared between early time points 1 to 3, but some (OTU-6, OTU-22, and OTU-46 unclassified *Enterobacteriaceae*) were also shared during later time points 4 and 5.

Four of the 50 most abundant OTUs, OTU-20, OTU-21 (both unclassified *Ruminococcaceae*), OTU-32 (unclassified *Clostridiales*), and OTU-48 (*Comamonas*), were shared between cow and calf faecal samples but only during early time points 1 to 3.

The colostrum and calf faecal microbiota shared 10 out of the 50 most abundant OTUs: OTU-1 and OTU-3 (unclassified *Enterobacteriaceae*), OTU-2 and OTU-13 (*Enhydrobacter*), OTU-4 and OTU-18 (*Acinetobacter*), OTU-28 (*Pseudomonas*), OTU-33 (*Propionibacterium acnes*), OTU-47 (*Stenotrophomonas*), and OTU-48 (*Comamonas*). All of these OTUs were only shared between colostrum and early calf faecal sampling time points 1 to 3, not with time points 4 and 5.

Calf mouth and calf faecal microbiota shared several of the 50 most abundant OTUs, such as OTU-4 (*Acinetobacter*), OTU-6 (unclassified *Enterobacteriaceae*), OTU-11 (*Sphingomonas*), OTU-18 (*Acinetobacter*), OTU-19 (*Solibacillus*), OTU-20 and OTU-21 (unclassified *Ruminococcaceae*), OTU-28 (*Pseudomonas*), OTU-30 (*Acinetobacter lwoffii*), OTU-32 (unclassified *Clostridiales*), OTU-33 (*Propionibacterium acnes*), OTU-47 (*Stenotrophomonas*), and OTU-48 (*Comamonas*).

**Fig 5:**
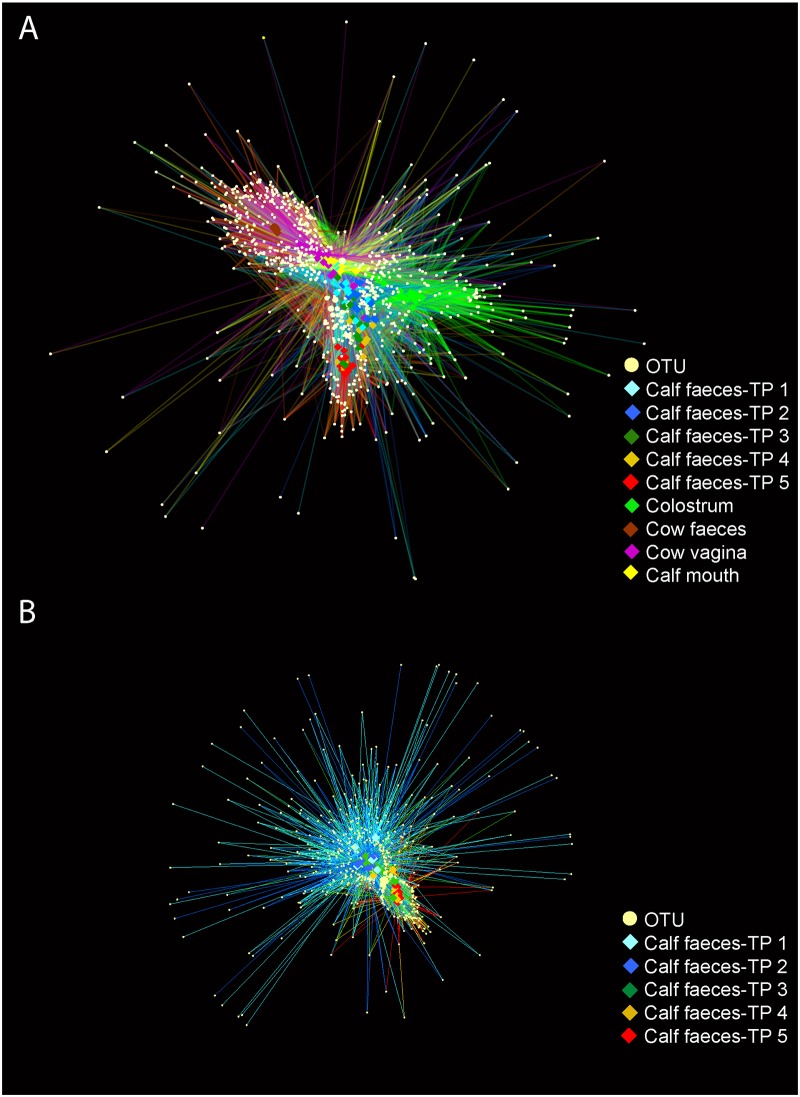
OTU sharing network. Light-yellow circular nodes represent the different OTUs (more abundant than 0.01% overall) and the size of each node is proportional to their relative abundance. The diamond shaped nodes represent the samples and are colored regarding the different types of samples. OTUs and the samples they belong to are connected by colored lines corresponding to the type of the sample. TP = time point, TP 1 = 0.5 hours *post natum* (*p*.*n*.), TP 2 = 6 hours *p*.*n*., TP 3 = 12 hours *p*.*n*., TP 4 = 24 hours *p*.*n*., TP 5 = 48 hours *p*.*n*.

Connections between samples can be visualized as an OTU network shown in Fig 5. In Fig 5A all samples are represented, and three edges that harbor cow faeces, colostrum or late-time point calf faeces, respectively, can be observed. The center of the network, which is occupied by all the samples and OTUs with more connections with other elements in the network, revealed close relationships between the calf faeces samples (at early time points), calf mouth samples, and cow vagina samples. As time increased calf faecal samples tended to cluster together along one of the edges of the network. The evolution of calf faecal microbiota can be observed in Fig 5B where only calf faecal samples are represented. Time points 1 and 2 showed a high number of low abundant OTUs, most of which were only found in these two time points. Microbial communities develop and establish throughout time, and samples with a lower number of high abundance OTUs clustered together.

## Discussion

The intestinal microbiota has an important effect on the development of the newborn`s immunity and metabolism, especially energy metabolism [25], and consequently on health, performance, and overall welfare. Hence, in our study we examined the early calf faecal microbiota and its possible sources.

In the present study, we choose to examine faeces as this is a non-invasive and practicable method to sample animals repeatedly without euthanisation. It should however be noted, that faecal samples do not fully represent the microbiota of particularly the upper gastro-intestinal tract. It is known that the microbiota of gastro-intestinal luminal content and of mucosal or epithelial surfaces of the gastro-intestinal tract differs [3, 26]. Consequently, the presented results of faecal samples examinations do not fully reflect early colonization processes of mucosal or epithelial gastro-intestinal tract surfaces.

### The calf microbiota is highly variable during the first 48 hours of life

In the present study we provide detailed analyses of the faecal calf microbiota during the first two days of life. Species richness and diversity, as well as the number of observed OTUs, differed significantly within calf faecal samples and were significantly lower than in adult cattle. An increase in species richness and diversity with increasing age has been described in other studies [5, 27–29]. In contrast, during the short time period of two days *p*.*n*., species richness, diversity, and number of observed OTUs decreased significantly between 6 and 24 hours *p*.*n*. in our study. A decrease in mean Chao1, Shannon diversity index, and observed OTUs, during the first one to two days *p*.*n*. was also seen in calves in the studies of Alipour et al. [2] and Yeoman et al. [3], although their numbers were considerably lower than in our study. Similar results were obtained in humans and mice [30–32]. Possible explanations given by these authors are selective effects from the environment, especially milk, and a higher diversity immediately *p*.*n*. caused by prenatal colonisation.

As shown by qPCR, copy numbers increased significantly between 6 and 24 hours *p*.*n*., as has also be seen in calves [2] as well as in humans [33].

In general, the calf faecal microbiota was clearly dominated by *Proteobacteria*, followed by *Firmicutes*, *Actinobacteria*, and *Bacteroidetes*. In contrast to *Proteobacteri*a and *Firmicutes*, *Actinobacteria* and *Bacteroidetes* were only present during early time points (0.5 and 6.0 hours *p*.*n*.). These findings are comparable to the findings in studies in newborn calves and humans [2, 3, 34]. During later time points and in adult cattle *Proteobacteria* usually decrease and *Firmicutes* and *Bacteroidetes* dominate the faecal microbiota [2, 3, 28, 29, 35, 36]. At OTU level, the calf faecal microbiota was dominated by unclassified *Enterobacteriaceae* OTUs which were found in all samples. Similarly, the faecal microbiota of newborn calves was dominated by unclassified *Enterobacteriaceae* in the studies of Alipour et al. [2] and Yeoman et al. [3]. We identified several OTUs which were only found during the first two to three sampling time points (immediately after birth to 12 hours *p*.*n*.) and OTUs which were found only during the later time points 4 and 5 (24 and 48 hours *p*.*n*.). This indicated a change in the most abundant OTUs between 6 and 12 hours *p*.*n*. and after 24 hours *p*.*n*. These findings are similar to the finding of Mayer et al. [37] that the main change in the bacterial flora in calf faeces was detected between 12 and 24 hours *p*.*n*. Generally, the microbiota seems to become more stable from earlier to later time points in humans and cattle [27, 38].

### Microbiota of the cow (vagina, faeces, and colostrum) revealed clear dominance of OTUs belonging to one family and genus

In the present study, unassigned *Ruminococcaceae* OTUs were most abundant in cow vaginal samples. This is similar to the findings of Clemmons et al. [39] in bovine vaginal samples *post partum* (*p*.*p*.). Furthermore, Karstrup et al. [40] examined endometrium and placentomes of pregnant cows and described a high abundance of bacteria belonging to the family *Ruminococcaceae*. Laguardia-Nascimento et al. [41] and Yeoman et al. [3] detected that, among others, *Ruminococcus* belonged to the vaginal core microbiota in cattle.

Similarly, the cow faecal microbiota was clearly dominated by unclassified *Ruminocaccaceae*. This high abundance of *Ruminococcaceae* in cow vaginal and faecal samples indicates similarity between these two sampling sites. Overall, cow vaginal and faecal microbiota shared 30.3% of their OTUs. A similarity can also be seen in OTU sharing networks (Fig 5A). Jeon et al. [42] also detected similarities in the cow vaginal, faecal, and blood bacterial composition immediately *p*.*p*., suggesting some haematogenic spread from the intestines to the reproductive tract.

*Enhydrobacter* was the most abundant OTU found in colostrum samples of our study. It should be noted that the taxonomy of the genus *Enhydrobacter* is currently under discussion, and it has been suggested that *Enhydrobacter* should be placed in the *Alphaproteobacteria* [43]. In contrast to our study, bovine colostrum samples of other studies were dominated by *Lactococcus*, *Staphylococcus*, *Pseudomonas*, *Streptococcus*, and *Escherichia* [3, 44]. In human colostrum samples, *Staphylococcus* and *Streptococcus* were also regularly detected, as well as *Enhydrobacter*, although other bacteria seem to be of higher abundance in human colostrum [45–48]. In bovines, *Enhydrobacter* were so far only described in cheese made from raw cow’s milk [49–51].

### Potential sources of the microbiota of newborn calves during the first 48 hours of life include the vaginas of their mothers and other unexamined factors e.g. environment

To identify possible inoculation routes for the calf microbiota, we analysed OTUs shared between samples. The calf microbiota during the first two days of life was clearly distinct from the dam’s faecal microbiota as well as from microbial communities from adult cattle from other studies which were dominated by OTUs assigned to *Ruminococcaceae*, *Prevotella*, *Paraprevotellaceae*, *Lachnospiraceae*, *Turicibacter*, *Bacteroidaceae*, *Succinivibrio*, and *Clostridiaceae* [8, 28, 35, 36]. This finding is not surprising as a newborn calf is fed by liquid feed only and has not developed a functional rumen yet. Similarly, Alipour et al. [2] found differences between the newborn calf and cow faecal microbiota that became more similar within 7 days after birth.

All calves in this study were fed with their mother’s colostrum within two hours *p*.*n*. Our results indicate that the colostrum microbiota is distinct from the calf and cow faecal microbiota and thus most likely does not play an important role as inoculation source for calf microbiota during the first two days of life. Potentially, colostrum has a beneficial effect on the calf gastrointestinal microbiota later in life, as suggested in the study of Yeoman et al. [3] or may influence the intestinal epithelial microbiota, what has not been examined in the present study. The most abundant colostrum OTUs of our study such as *Enhydrobacter* are not known to be abundant in the cattle digestive tract. In agreement with current knowledge, the main benefit of feeding first colostrum to calves within the first hours *p*.*n*. is the provision of immunoglobulins [52].

All calves in our study were delivered vaginally. An inoculation of newborns with the microbiota of their mother’s birth canal has been described in humans [13]. Newer studies suggest that in humans the vaginal microbiota is not a source of the infant`s faecal microbiota [53, 54]. In cattle the results are also contradictory [2, 3]. In contrast to the colostrum and cow faecal microbiota in our study, the cow vaginal and the calf faecal microbiota were more similar (as can be seen in OTU sharing networks, Fig 5A) and shared overall 2,240 OTUs, suggesting that some of the calf faecal microbiota may derive from inoculation from the birth canal.

Another possible inoculation source of the calf microbiota might arise from the calf licking the vagina or the environment during and immediately after birth. A few OTUs showed highest abundance in the calf mouth samples taken within 0.5 hours *p*.*n*. such as OTU-4 (*Acinetobacter*) and OTU-19 (*Solibacillus*) and were also detected in faecal calf and cow samples, but their abundance in faecal samples decreased and they could not be detected further. We thus assume that the calf’s mouth is not a significant source of inoculation of the gastrointestinal tract microbiota during the first two days of life.

Interestingly, we identified a number of OTUs which showed intermediate abundances which were only found in calf faecal samples. These OTUs can derive from other, potentially environmental, sources that were not sampled in our study or might represent OTUs that were already present in the gastrointestinal tract before birth. A possible inoculation of the fetus with bacteria before birth has been suggested in humans, mice, and cattle [2, 48, 55–57]. Based on the presence of a diverse microbiota and comparatively high copy numbers (median: 8.74×10^3^ copy numbers) in a relatively short time period after birth (0.5 hours *p*.*n*.) we believe that an inoculation of these OTUs from the vagina, colostrum, or environment is rather unlikely. The fact that these OTUs were generally not present in the calf mouth also indicates that these OTUs might already have been present in the fetus before birth. Further studies are nevertheless necessary to elucidate this hypothesis.

Summarizing the potential sources of the early faecal microbiota of newborn calves reveal that the bacteria found in the first calf faecal samples (sampled within 0.5 h *p*.*n*.) can have an intra-uterine origin, as it would be unlikely that microbes from the vagina of the mother, colostrum, or environment are detected in faecal samples that early. Some of the calves`faecal microbes detected can be explained by vaginal transmission during birth. Several OTUs common to vagina and calf mouth were also found in calf faeces during early sampling time points 1 to 3 (0.5 to 12 hours *p*.*n*.). The fact that the majority of these OTUs were not detected at later time points suggests that once the calves are exposed to the environment and to the milk diet their gut microbiome shapes. The fact that not many OTUs have been shared by colostrum and calf faeces may indicate that colostrum does not play an important role as a source of intestinal microorganisms, but is of importance from an immunological point of view.

## Conclusion

The calf faecal microbiota was highly variable during the first 48 hours *p*.*n*. The calf faecal microbiota during the first two days of life was clearly distinct from the mother’s faecal microbiota and colostrum. In contrast, the cow vaginal and the calf faecal microbiota were more similar, suggesting that some of the calf faecal microbiota may derive from inoculation from the birth canal during birth. Other sources not examined in the present study could be the environment and the uterus *ante natum*.

## Supporting information

S1 Table The 50 most abundant OTUs per sampling group, median (Med) relative abundance (%) and 25 and 75% percentile (%) are listed. OTU consensus taxonomy is based on the GreenGenes 16S rRNA gene database.
(XLSX)

S1 Fig Median relative abundance of phyla in the different samples and significant (*P*≤0.05) differences to other samples. TP = time point, TP 1 = 0.5 hours post natum (*p*.*n*.), TP 2 = 6 hours *p*.*n*., TP 3 = 12 hours *p*.*n*., TP 4 = 24 hours *p*.*n*., TP 5 = 48 hours *p*.*n*.
(TIF)
