# Supplementary figures and images for: Microbiota of newborn calves and their mothers reveals possible transfer routes for newborn calves’ gastrointestinal microbiota
Source: PLoS One. 2019 Aug 1;14(8):e0220554. doi: 10.1371/journal.pone.0220554 (PMC6675284; doi:10.1371/journal.pone.0220554)

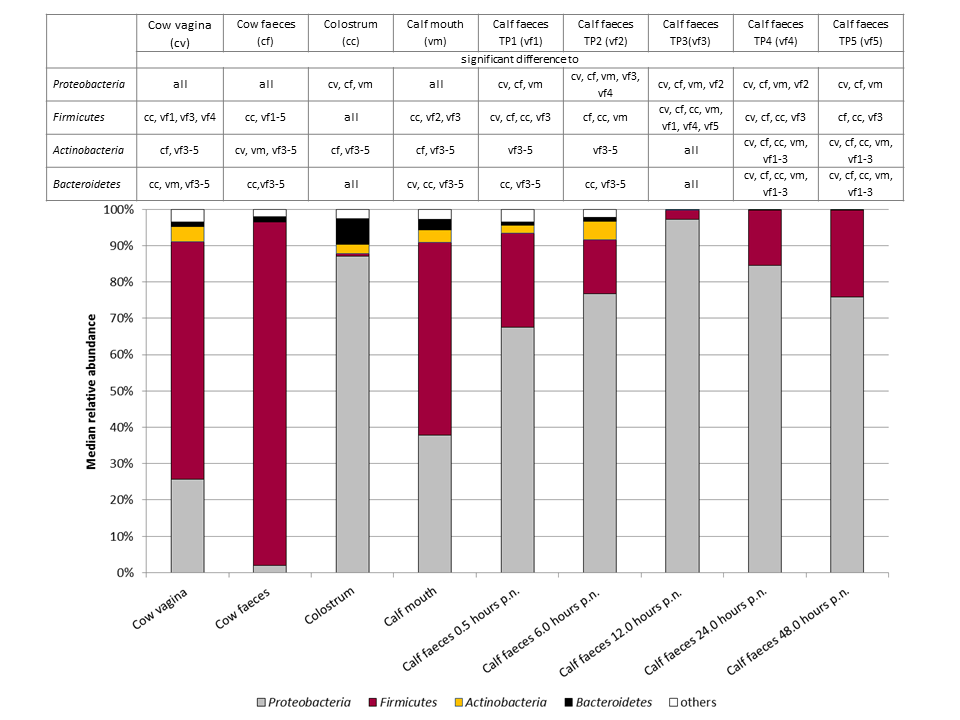

Supplement: S1 Fig — TP = time point, TP 1 = 0.5 hours post natum (p.n.), TP 2 = 6 hours p.n., TP 3 = 12 hours p.n., TP 4 = 24 hours p.n., TP 5 = 48 hours p.n. (TIF) [file pone.0220554.s002.tif]
